# Illuminating the harvest: the regulatory effects of LEDs on pigment accumulation in various food crops

**DOI:** 10.1007/s12298-025-01596-0

**Published:** 2025-05-26

**Authors:** Zhang Yaoyuan, Nyok-Sean Lau, Sreeramanan Subramaniam

**Affiliations:** 1 Centre for Chemical Biology (CCB), Universiti Sains Malaysia (USM), 11900 Bayan Lepas, Penang, Malaysia; 2 School of Biological Sciences, Universiti Sains Malaysia (USM), 11800 Georgetown, Penang, Malaysia; 3 Institute of Nano Optoelectronics Research and Technology, Universiti Sains Malaysia (USM), 11900 Bayan Lepas, Penang, Malaysia; 4 BioLED Technology Sdn Bhd, Universiti Sains Malaysia (USM), 11900 Bayan Lepas, Penang, Malaysia

**Keywords:** Food plants, LED, Pigments, Non-coding RNA, Metabolite

## Abstract

Food plants provide vital nutrients for humans and are the basis for their survival. The pigments in food plants not only improve their sensory value, but also increase their medicinal and nutritional value, which has a positive effect on human health. Light can influence the accumulation of pigments in food plants, and different light qualities, intensities and cycles have different effects on the accumulation of different pigments. For example, blue light can promote the production of chlorophyll and anthocyanins, while red light favours the accumulation of carotenoids. With the development of plantation agriculture, LED light sources are gradually being used for the market-orientated production of food crops. In recent years, research has shown that non-coding RNAs such as miRNA and lncRNA significantly influence the process of light-regulated pigment accumulation. Non-coding RNA can modulate the expression of genes related to pigment metabolism and thus influence pigment accumulation. Investigating the effect of LED light on the expression of non-coding RNA can further elucidate the molecular mechanism of light regulation of pigment accumulation and provide a new theoretical basis for the precise regulation of pigment accumulation. Therefore, we summarised the effects of LED light quality, intensity and period on pigments in food plants and elucidated the regulatory role of LED light on non-coding RNAs related to pigment metabolism in food plants, which theoretically supports the application of LED light sources in food plants.

## Introduction

Food crops are very important for agriculture and human health. They are crops grown for human consumption, including grains such as wheat, rice and maize, pulses such as soya beans and lentils, and various fruits, vegetables and root crops. Pigments are colourants produced by plants. They are broadly divided into two categories: fat-soluble pigments (like chlorophyll and carotenoids) and water-soluble pigments (like anthocyanins and flavonoids) (Aly and Borik 2023; Raja and Tennyson 2023; López-Cruz et al. 2023).

Pigments can be used in many areas, e.g. in the food industry, in pharmaceuticals, in cosmetics, etc. In the food industry, food processors often use pigments such as carotene to colour foods and enhance their attractiveness (Ayala-Zavala et al. 2011; Johnson 2014). Due to their diverse pharmacological effects, pigments are also used in the pharmaceutical sector. Lutein and zeaxanthin in maize and cucumber have the effect of reducing the risk of eye diseases and protecting the retina (Abdel-Aal et al. 2013). Pigments can also be used as raw materials for cosmetics. Anthocyanins, for example, are used in the manufacture of cosmetics (Cefali et al. 2019).

The metabolism of pigments in plants is controlled by many factors such as temperature, soil conditions, humidity, etc. In addition to these factors, light also plays a role. The amount and quality of light a plant receives have a dramatic effect on its pigment composition. For example, red light alone resulted in a significantly lower carotenoid content in tomatoes with a 10:01 ratio of red to blue light (Naznin et al. 2019).

Driven by global urbanisation and decreasing arable land, the cultivation of plants with artificial light sources indoors is gradually increasing. Originally, incandescent, fluorescent and high-pressure sodium lamps were used as light sources for irradiating food crops, but due to the shortcomings of conventional light sources such as incomplete spectrum, low efficiency, high heat generation and short lifetime, they were gradually replaced by Light Emitting Diode light sources (LED light) (Massa et al.; Long et al. 2015), research and summaries on the effects of LED light on food crops mainly focus on the effects on morphogenesis and photosynthetic rate (Johkan et al. 2010; Tewolde et al. 2016), but a systematic summary on the influence of LED light on the secondary metabolism of food plants, especially the metabolism of pigments, is still lacking (Ouzounis et al. 2015b; Alrifai et al. 2019). Therefore, it is crucial to investigate the effects of LED light on plant pigment metabolism.

Non-coding RNA (ncRNA) is a general term for ribonucleic acid that is not encoded as a protein but directly performs biological functions in the form of RNA. In plants, it mainly comprises (i) small ncRNA, namely microRNA (miRNA) and small interfering RNA (siRNA), and (ii) long non-coding RNA. They have a significant impact on the modulation of plant gene expression, maintenance of genome integrity and long-distance signalling (Khraiwesh et al. 2012). However, research on non-coding genes in plants is still at an early stage (Zhao et al. 2022), and an overview of the regulation of non-coding gene expression by light, especially LED light is lacking.

**Fig. 1:**
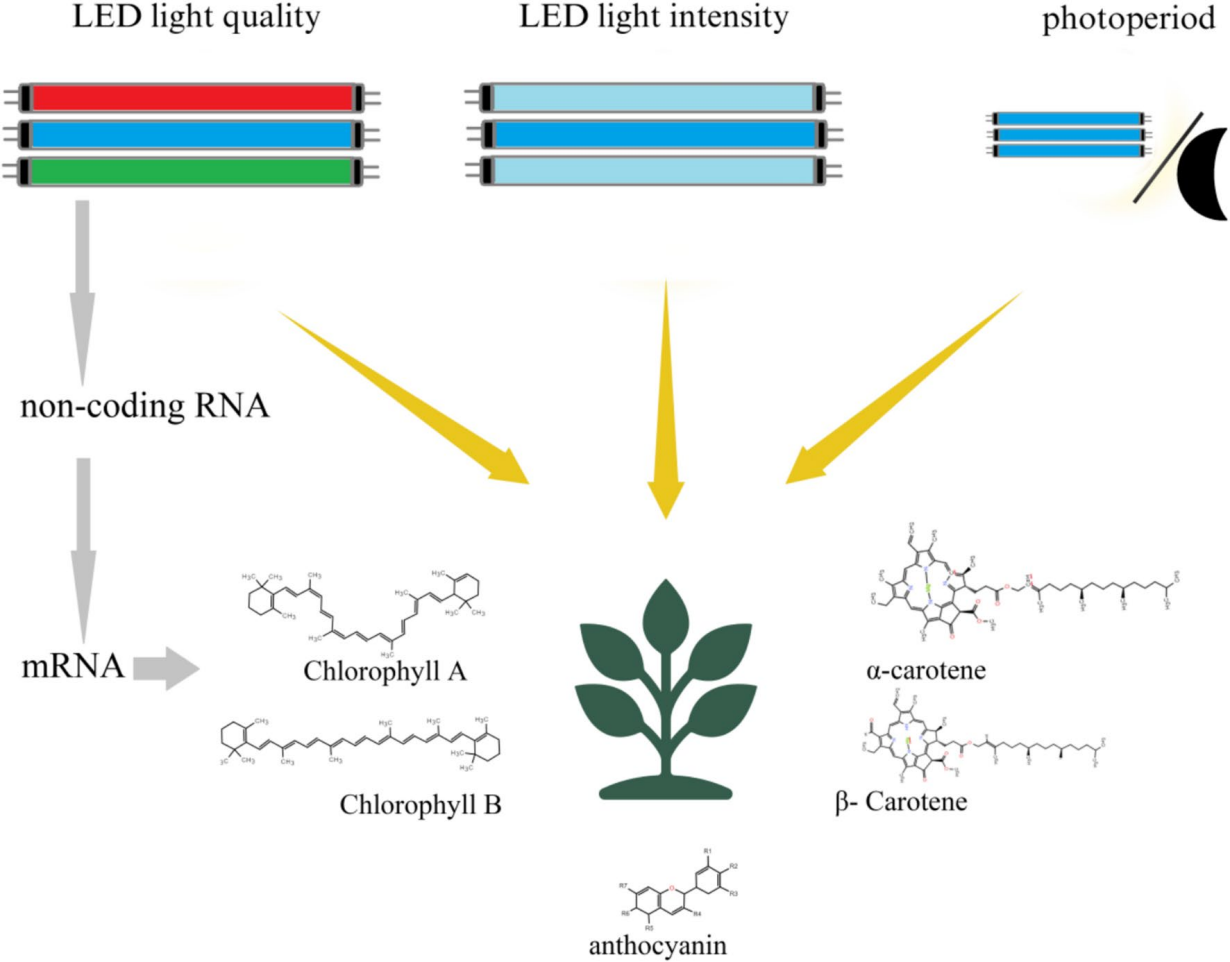
LED light affects the common pigment metabolism in food crops

This review provides a detailed overview of the effects of LED light on pigment metabolism and the expression of non-coding genes in food crops (Fig. 1) and provides theoretical support for optimising indoor growing practices and improving the quality of food crops.

## Concept of LED light quality, intensity and photoperiod

LED light quality refers to the spectral distribution emitted by LED sources (Zhang et al. 2019). Different wavelengths correspond to different light colours: far-red (1000–700 nm), red (600–700 nm), yellow-green (500–600 nm), blue (400–500 nm), Ultraviolet A (UV-A) (315–400 nm), and Ultraviolet B (UV-B) (280–315 nm). Blue LEDs play an important role in chloroplast development, chlorophyll formation and stomatal opening (Zhang et al. 2018a, b, c). A combination of blue and red LEDs promotes seedling growth and development (Li et al. 2012).

Luminous intensity refers to the intensity of the light emitted by a light source (Guedes et al. 2023). The intensity of LED lighting significantly affects the growth and quality of plants. For example, low light intensity cannot maintain plant growth due to insufficient energy supply, Supplemental LED lighting is required to ensure crop yield and nutritional quality (Yorio et al. 2001). Although high light intensity enhances photosynthesis, excessive light intensity can induce photoinhibition and cause damage to plant cells (Zheng et al. 2015).

The photoperiod refers to the length of time a plant is exposed to light (Roeber et al. 2022). LED light cycles significantly impact plant physiology, influencing flowering and seed germination. Adjusting photoperiod can modulate plant growth rates from seedling to maturity, potentially shortening vegetable growth cycles (Gawande et al. 2023). The photoperiod also affects the accumulation of metabolites in plants, including sugars, phenols and amino acids, and thus influences the quality and yield of the plants (Xu et al. 2020).

## LED light quality affects plant growth and development by influencing pigment metabolism

Light quality is an important regulatory factor that influences plant seed germination, light morphogenesis, response to shade avoidance, flowering and senescence as well as physiological and metabolic responses (Xu et al. 2005). Different light quality modulates plant growth and development by regulating pigment metabolism in plants (Abidi et al. 2013). For example, light quality acted as a regulator of pigment content in strawberry leaves and altered their morphology (Posada et al. 2014). In peppers, violet light led to changes in the carotenoid composition, which in turn influenced the colour of the pepper leaves (Liu et al. 2022). Supplementation with blue light increased the content of lycopene, a series of carotenes including β-carotene, in fruits during fruit ripening and accelerated colour development (Wang et al. 2021b).

LED light not only directly regulates the growth and development of food crops (Dong et al. 2014; Suh et al. 2020) but also influences the physiological processes of cereal plants by affecting pigment metabolism. Physiological processes refer to a series of ordered and typically interacting biochemical reactions that occur in an organism and that affect the structure, function and metabolism of the organism. In the context of plant growth and development, physiological processes include photosynthesis, respiration, hormone regulation, enzymes, mass transport, and the synthesis and decomposition of metabolites (Kozlowski and Pallardy 1997).

Blue LED light influences leaf colouration by promoting the accumulation of carotenoids and anthocyanins (Li and Kubota 2009). Red and blue LED lights promoted the synthesis of anthocyanins and carotenoids in Guzmania lingulata, resulting in improved light absorption capacity (Keyser et al. 2019). A combination of red and blue LED light increased chlorophyll content in Chinese cabbage, peppers, strawberries and broccoli (Avercheva et al. 2010; Samuolienė et al. 2010; Baniekal et al. 2012; Kopsell et al. 2014), and chlorophyll concentration could directly influence the photosynthetic rate (Hoober and Eggink 1999). Treatment with red, blue and red-blue light significantly increased the total anthocyanin content in strawberry fruit, resulting in earlier fruit colouration (Zhang et al. 2018b). Additional blue light or red light in combination with blue light increased the content of lycopene, β-carotene, α-carotene and γ-carotene, thus accelerating fruit colouration (Wang et al. 2021a). Red light and far-red radiation can activate photoreceptors in strawberries, inhibit the function of specific functional proteins, thereby triggering the upregulation of flavonoid gene expression, affecting the content of flavonoids and other secondary metabolites in strawberries, and ultimately affecting fruit development (Warner et al. 2021). It is therefore necessary to investigate the effects of LED light quality on the pigment metabolism of food plants.

## The effect of blue LED light on pigment content in food crops

Blue LED light has been shown to promote the accumulation of active compounds such as flavonoids and anthocyanins in various food plants. For example, an increase in anthocyanin accumulation was observed in *Fagopyrum tataricum* under blue LED irradiation (Thwe et al. 2014). Blue light significantly upregulates the expression of phenylalanine ammonia-lyase (FtPAL), a key gene for flavonoid biosynthesis, and anthocyanidin synthase (FtANS), which is essential for anthocyanin accumulation. Short-term irradiation with blue LED light also led to an increased content of pigments such as β-carotene and zeaxanthin in the bud tissue of broccoli (Kopsell and Sams 2013). Previous studies have shown that blue light irradiation significantly upregulates the expression levels of two isoforms of phytoene synthase (PSY), ξ-carotene desaturase (ZDS) and lycopene β-cyclase (LCYb) in citrus yellow leaves. Based on these results, the authors hypothesize that blue light could enhance the expression of key enzyme genes in the carotenoid biosynthetic pathway.

Blue light has been found to increase anthocyanin content in strawberries and grapes and upregulate the activity of enzymes related to flavonoid metabolism in strawberries and grapes (Kondo et al. 2014; Zhang et al. 2018a, b, c). For example, blue light can increase the activity of enzymes such as PAL, cinnamate 4-hydroxylase (C4H), 4-coumarate: CoA ligase (4CL), chalcone synthase (CHS), flavanone-3-β-hydroxylase (F3H) and dihydroflavonol 4-reductase (DFR) in the plant. These enzymes are not only involved in the biosynthesis of anthocyanins, but also play a crucial role in the biosynthetic pathways of various flavonoids, including flavonols, flavones and isoflavones.

In the study by Wang et al. (2022), in which soya sprouts were irradiated with infrared, blue, green and ultraviolet light, it was found that blue light promotes the accumulation of isoflavones and antioxidant activity the most. Previous studies have shown that blue light can significantly increase the activity of PAL in callus cultures of *Saussurea medusa* and soya seedlings, thereby promoting the synthesis of flavonoids (Dexiu et al. 1999; Li et al. 2017), Ensminger and Schäfer (1992) indicated that blue light can promote PAL activity in plant cells such as parsley (Ensminger & Schäfer 1992). Under blue light, the upregulation of isoflavone synthase (IFS) and CHS expression leads to an increased content of the isoflavone monomer daidzein in the cotyledons. At the same time, blue light also upregulates the expression of ANS, a key gene for anthocyanin biosynthesis.

Blue LED lighting increased the anthocyanin content in red lettuce. Similarly, lettuce under blue LED light showed a significant increase in anthocyanin, lutein and β-carotene content (Ouzounis et al. 2015a, b). Irradiation with blue LED light stimulated the activity of the key enzyme PAL in red lettuce, which led to the synthesis of various pigments (Heo et al. 2012). Giliberto et al (2005) found that tomatoes accumulate a large amount of anthocyanins when exposed to blue light. Blue light induces an overexpression of the blue light receptor cryptochrome 2 (CRY2), which leads to an increased accumulation of anthocyanins in the leaves and other organs of the tomato. Li and Kubota (2009) also found that blue LED light increased the content of carotenoids in lettuce. The authors indicated that blue light stimulates the anthocyanin pathway in Gerbera hybrida by upregulating the expression of CHS and DFR genes (Meng et al. 2004). The concentration of carotenoids (lutein and β-carotene) in plants increases in response to blue light as a protective mechanism against the detrimental effects of high blue light intensities (Landrum and Bone 2001). In addition, blue light promoted the synthesis of betalanine and carotenoids most strongly in *Amaranthus tricolour* compared to darkness (Zhang et al. 2018a).

However, supplementation with blue LED light resulted in a remarkable decrease in chlorophyll B content and an increase in carotenoid content in broccoli microgreens (Kopsell and Sams 2013). The first step in the carotenoid biosynthetic pathway involves the condensation of two geranylgeranyl diphosphate molecules by PSY to form phytoene. Subsequent desaturation reactions, catalysed by phytoene desaturase (PDS) and ZDS, convert phytoene to lycopene. Lycopene is then cyclised by LCYb and lycopene ε-cyclase to form various carotenoids. The cyclisation process can result in carotenoids with two β-rings (such as β-carotene, zeaxanthin, antheraxanthin, violaxanthin and neoxanthin) or one β-ring and one ε-ring (such as α-carotene and lutein). Blue light has been shown to significantly increase the mRNA levels of two PSY isoforms, ZDS and an isoform of LCYb in *Citrus flavedo*, suggesting that blue LEDs may regulate carotenoid content in broccoli microgreens by modulating the activity of carotenoid-related enzymes.

## The effect of red LED light on pigment content in food crops

Red LED irradiation promoted the accumulation of β-carotene in pea seedlings (Wu et al. 2007), increased the lycopene content in tomatoes (Xie et al. 2016) and significantly increased the anthocyanin concentration in kale shoots (Mizuno et al. 2011). Red light is hypothesized to increase carotenoid levels in pea seedlings by activating *Lycopersicon esculentum* elongated hypocotyl 5 (LeHY5) and repressing *Lycopersicon esculentum* constitutive photomorphogenesis 1-like (LeCOP1LIKE). However, it reduced the total anthocyanin content in Brassicaceae plants (Brazaityte et al. 2016b, a). Red LED light also increased the carotenoid concentration in greenhouse-grown cucumber seedlings (Wang et al. 2009), increased the content of lutein and β-carotene in kale by promoting their biosynthesis, and promoted the accumulation of chlorophyll A and chlorophyll B (Lefsrud et al. 2008). Mizuno et al. (2011) reported that red light promoted the accumulation of total anthocyanin content in “*red rookie*”.

Di et al. (2021) found that the content of chlorophyll A, chlorophyll B and carotenoids in eggplant was significantly increased after irradiation with red light compared to other types of light. Irradiation with red LED light improved the β-cryptoxanthin content in citrus fruits and the lycopene content in tomatoes (Ma et al. 2012; Xie et al. 2019). Red light irradiation upregulates the transcription of genes encoding CitPSY, CitPDS, CitZDS, CitLCYb1, CitLCYb2, CitHYb, and zeaxanthin (zea) epoxidase (CitZEP) in tomato fruits, thereby promoting the biosynthesis of β-cryptoxanthin in tomatoes. Xie et al. suggested that red light inhibits the expression of PIF proteins by enhancing PHY activity, thereby promoting PSY expression or inducing CRY expression, ultimately leading to an increase in lycopene content. Studies have found that red LED irradiation can extend the shelf life of broccoli at room temperature by inhibiting yellowing and suppressing the expression of chlorophyll degradation genes chlorophyllase I(BoCLH1), chlorophyllase II (BoCLH2), chlorophyllase III (BoCLH3), and pheophorbide a oxygenase (BoPAO), thus maintaining higher chlorophyll levels (Jiang et al. 2019).

## The effect of red and blue combined light on the pigment content in food crops

In comparison to additional white light, cabbage sprouts under red and blue combined light showed an increased carotenoid content in 15 days (Metallo et al. 2018). In the work of Nguyen et al. (2021), the use of red-blue (4:1) mixed light significantly increased the photosynthetic pigments in spinach and improved its quality. This is because chlorophyll a and b primarily absorb blue and red light, respectively, with red light having a greater impact on photosynthesis. As the proportion of red light decreases, the content of photosynthetic pigments also decreases. Consequently, the photosynthetic pigment content under R4B1 treatment was higher than that under R5B2G3 and R1B1G1 treatments.

Irradiation with blue and red light in a 1:4 ratio induced the expression of genes encoding enzymes related to flavonoid synthesis in *Anoectochilus formosanus* and resulted in increased flavonoid accumulation (Gam et al. 2020). The highest expression levels of chi (chalcone isomerase) and fls (encoding flavonol synthase) genes under this light condition may lead to an increase in flavonoid content in *Anoectochilus formosanus*. When treated with red-blue light, the synthesis of cucumber pigments was significantly higher compared to treatment with monochromatic light (Kim et al. 2012). The synthesis of chlorophyll and carotenoids in spinach was upregulated when irradiated with red and blue light (Huang et al. 2018). Compared to monochromatic red or blue light, red and blue light increased the content of chlorophyll and anthocyanins in basil (Lobiuc et al. 2017) This is due to the fact that blue light improved the gene expression of magnesium chelatase (MgCH), glutamyl-tRNA reductase (GluTR), and ferrochelatase (FeCH), which are involved in chlorophyll synthesis, while high-throughput red light led to a decrease in 5-aminolevulinic acid, a tetrapyrrole precursor required for chlorophyll synthesis.

At the same time, red light increased the carotenoid content, and the addition of blue light further enhanced the production of carotenoids. Tomatoes illuminated with red light (88% of intensity) and blue light (12% of intensity) showed higher chlorophyll concentrations (Ouzounis et al. 2016).

## The effect of UV light on pigment content in food crops

Although studies have shown that UV radiation disrupts the photosynthetic apparatus of crop plants, damaging plant tissue and impairing productivity (Maiti and Satya 2014), UV light can also act as a stress signal by triggering the production of various secondary metabolites that increase disease resistance (Meyer et al. 2021). Among these secondary metabolites, pigments play a crucial role in plant adaptation to UV radiation due to their antioxidant potential (Takshak and Agrawal 2019).

Li and Kubota (2009) discovered that the content of anthocyanins, lutein and β-carotene in lettuce (*Lactuca sativa* L. cv. Red Cross) increased after exposure to UV-B and UV-A light. The author suggests that ultraviolet light with a shorter wavelength could increase anthocyanin concentration more effectively. When UV-B irradiation was increased, the content of both light-harvesting pigments, such as chlorophyll and photoprotective plant pigments such as carotenoids increased. UV-B reduced the pigment content in wheat but promoted the accumulation of flavonoids in *Arabidopsis thaliana* (Stracke et al. 2010). When exposed to ultraviolet radiation, flavonoids can absorb part of the UV energy and thus reduce the damage to plant cells caused by UV radiation. This is why flavonoids are also known as natural UV filters. The significant decrease in chlorophyll content caused by UV-B treatment was attributed to the inhibition of its biosynthesis and the degradation of its precursors. Jiao et al (2016) reported that UV-B light with a wavelength of 313 nm increased the content of isoflavones in soya bean sprouts. UV-B radiation promoted the biosynthesis of endogenous abscisic acid (ABA) and isoflavones in soya bean shoots. Simultaneously, ABA activated the protein and gene expression of CHS and IFS in soybean shoots through cyclic ADP-ribose (cADPR) and Inositol 1,4,5-trisphosphate (IP3) induced by UV-B radiation and enhanced the accumulation of isoflavones via a Ca^2+−^mediated signalling pathway. While UV-A increased the total chlorophyll and anthocyanin content of broccoli (Gongheng et al. 2024). UV-A radiation promoted the electron transport chain in the photosynthetic system of broccoli, thereby stimulating the production of more chlorophyll through photosynthesis.

Similarly, UV light can significantly increase the accumulation of flavonoids in fruits, often by upregulating gene expression associated with flavonoid metabolism (Zoratti et al. 2014). Studies have shown that the content of flavonoids, especially quercetin and kaempferol glycosides, was increased in grapes exposed to UV-B radiation compared to non-irradiated controls. This upregulation coincides with a corresponding increase in the expression of flavonol synthase 4 (VvFLS4), a gene that is crucial for flavonol metabolism in grapes (Liu et al. 2015). In apples, UV-B radiation increased the expression of CHS, DFR, F3H, leucoanthocyanidin dioxygenase (LDOX) and UDP-glucose flavonoid 3-O-glucosyltransferase (UFGT) genes in flavonoid metabolism (Ubi et al. 2006) and induced a positive response of enzymes related to flavonoid metabolism. UV light stimulates the production of nitric oxide (NO) in plants, which in turn activates guanylate cyclase (GC) and promotes the synthesis of cyclic guanosine monophosphate (cGMP). The elevated cGMP concentration leads to increased levels of inositol IP3, glycogen synthase kinase-3 (GSK-3), and gamma-aminobutyric acid (GABA), thereby promoting the biosynthesis of isoflavones (Jiao and Liu 2021).

## The influence of other coloured lights on the pigment content in food crops

In studies in which cherry tomato seedlings were irradiated with different qualities of light, the results showed that the concentration of flavonoids and antioxidant capacity in the seedlings varied from high to low under blue, red, green and white light (Liu et al. 2010).

The chlorophyll A content of wheat under complementary red and far-red LED light was significantly lower than under combined red-blue light (blue:red = 3:1 and blue:red = 1:5), while the chlorophyll B content showed no significant difference (Samuoliene et al. 2012). The combination of red and far-red LED irradiation increased the total anthocyanin content in lettuce plants (Li and Kubota 2009). In addition, Samuolienė et al. (2017) reported increased levels of carotenes and xanthophylls in young lettuce illuminated with a combination of white LED light supplemented with red and far-red LEDs compared to illumination with white LEDs alone.

Baby leaf lettuce showed a higher total anthocyanin content when grown under green LED irradiation compared to red LED irradiation (Samuoliene et al. 2012). Zhang and Folta (2012) found that anthocyanin synthesis induced by blue light can be reversed by green light. Similarly, green LED light was found to reduce blue light-induced anthocyanin accumulation in sprouting broccoli (Kopsell et al. 2014). Under green illumination, the neoxanthin content in red pakchoi microgreen increased by 5%, while it was significantly reduced by yellow light (Brazaityte et al. 2016b, a). Ban et al. (2019) investigated the effects of different light qualities on the quality and growth of pea shoots. The results showed that the quality of green light improved the carotenoid indices of pea shoots compared to the control group (Ban et al. 2019). Green light treatment also significantly reduced the lycopene content in tomato fruits (Xie et al. 2016).

Yellow LED light (595 nm) was able to increase the total content of carotenoids in tatsoi microgreen by 16% (Brazaityte et al. 2016b, a). Compared to white light, treatment with yellow light significantly increased the lycopene content in tomatoes (Xie et al. 2016). Conversely, studies by Xu et al. (2007) showed that yellow light reduced the carotenoid content of lettuce leaves compared to white light. The combination of yellow and blue LED light promoted the content of anthocyanins in Brassicaceae and beetroot microgreens, while orange LED light showed the opposite effect (Brazaityte et al. 2016b, a). Subsequently, Shao et al. (2021) discovered that post-harvest treatment with violet light can significantly increase lycopene content in tomato fruits. This result suggests that irradiation with violet light could be a promising strategy to increase the nutritional value of tomatoes. Table 1 presents the specific regulatory effects of various light qualities on grain crops.

**Table 1:** Modulatory effects of different LED lights on biosynthesis of pigment in food crops

No	Species	LED light	Modulation	References
1	Kale	Blue	Lutein, β-carotene, Chlorophyll ↑	Lefsrud et al. (2008)
2	*Fagopyrum tataricum*	Blue	Anthocyanin ↑	Thwe et al. (2014)
3	Broccoli	Short-duration	β-carotene, violaxanthin, total xanthophyll cycle pigments, glucoraphanin ↑	Kopsell and Sams (2013)
4	Grape	Blue	Anthocyanins ↑	Kondo et al. (2014)
5	Soybean	Blue	Isoflavones ↑	Wang et al. (2022)
6	Red lettuce	Blue	Anthocyanin ↑	Ouzounis et al. (2015a, b)
7	Lettuce	Blue	Anthocyanin, lutein and beta-carotene ↑	Ouzounis et al. (2015a, b)
8	Tomatoes	Blue	Anthocyanins ↑	Giliberto et al. (2005)
9	Lettuce	Blue	Carotenoid ↑	Li and Kubota (2009)
10	*Amaranthus tricolor*	Blue	βlaine and carotenoids ↑	Zhang et al. (2018a, b, c)
11	Broccoli microgreens	Blue	Chlorophyll b ↑	Kopsell and Sams (2013)
12	Pea	Red	β-carotene ↑	Wu et al., (2007)
13	Tomatoes	Red	Lycopene ↑	Xie et al. (2016)
14	Kale	Red	Anthocyanin ↑	Mizuno et al. (2011)
15	Brassicaceae	Red	Anthocyanin ↓	Brazaitytė et al., (2016a; b)
16	Cucumber	Red	Carotenoid ↑	Wang et al. (2009)
17	Kale	Red	Lutein, β-carotene, chlorophyll a, chlorophyll b ↑	Lefsrud et al. (2008)
18	Cabbage	Red	Anthocyanin ↑	Mizuno et al. (2011)
19	Eggplant	Red	Chlorophyll a, chlorophyll b, carotenoids ↑	Di et al. (2021)
20	Citrus	Red	β-cryptoxanthin ↑	Ma et al. (2012)
21	Tomatoes	Red	Lycopene ↑	Xie et al. (2019)
22	Cabbage	Red & Blue	Carotenoid ↑	Metallo et al. (2018)
23	Spinach	Red & Blue	Chlorophyll A, Chlorophyll B, Carotenoids ↑	Nguyen et al. (2021)
24	Lettuce	UV-B,UV-A	Lutein and β-carotene ↑	Li and Kubota (2009)
25	*Arabidopsis thaliana*	UV-B	Flavonoids ↑	Stracke et al. (2010)
26	Soybean	UV-B	Isoflavone ↑	Jiao et al. (2016)
27	Broccoli	UV-A	Chlorophyll ↑	Moreira-Rodríguez, et al., (2017)
28	*Fagopyrum tataricum*	UV	Carotenoid ↑	Thwe, et al. (2014)
29	Grapes	UV-B	Flavonol ↑, the expression of flavonol metabolism gene VvFLS4 was reduced	Liu et al. (2015)
30	Apple	UV-B	The expression of CHS, DFR, F3H, LDOX and UFGT genes in the flavonoid pathway was reduced	Ubi et al. (2006)
31	Lettuce	Red & Far red	Anthocyanin ↑	Li and Kubota (2009)
32	Tatsoi microgreen	Yellow	Carotenoid ↑	Brazaityte et al. (2016a, b)
33	Baby leaf lettuce	Green	Anthocyanin ↑	Samuolienė et al., (2012)
34	Mustard, Tatsoi	Yellow & Blue	Carotenoids ↑	Brazaityte et al. (2016a, b)
35	Lettuce	Red & far red & white	Carotenes and xanthophylls ↑	Samuolienė et al. (2017)
36	red pakchoi microgreen	Green	Neoxanthin ↑	Brazaityte et al. (2016a, b)
37	red pakchoi microgreen	Yellow	Neoxanthin ↓	Brazaityte et al. (2016a, b)
38	*Arabidopsis thaliana*	Green	Reverse blue-induced anthocyanin accumulation	Zhang and Folta (2012)
39	Broccoli	Green	Reverse blue-induced anthocyanin accumulation	Kopsell et al. (2014)
40	Tomato	Yellow	Lycopene content ↑	Xie et al. (2016)
41	Tomato	Green	Lycopene conten ↓	Xie et al. (2016)
42	Tomato	Purple	Lycopene content ↑	Shao et al. (2021)
43	Leaf lettuce	Yellow	Carotenoid ↓	Xu et al. (2007)
44	Pea	Green	Carotenoid ↑	Ban et al. (2019)
45	Wheat	Complementary red and far red light	Chlorophyll a ↓	Monostori et al. (2018)

^↑^^: The increase in metabolite content; ↓: The decrease in metabolite content^

## Effect of photoperiod on metabolism of pigment substances in food crops

Studies investigating the effects of different light cycles on purple leaf lettuce showed that alternating irradiation with red and blue light significantly increased the content of anthocyanins and total phenols in lettuce (Shao et al. 2021), and this might be related to PAL activity The effects of different photoperiods on Tartary buckwheat sprouts were investigated and it was found that the anthocyanin content was highest on the 2nd day of germination at a photoperiod of 12 h/12 h (day/night), and the expression of genes related to anthocyanin synthesis, FtDFR1 and FtANS, also increased, while the highest rutin content was observed after 4 days of light exposure at a photoperiod of 16 h/8 h (day/night) (Lu et al. 2019). The reason may be that a longer photoperiod (12–16 h per day) during the early germination stage (2–4 days) promotes the expression of genes related to rutin metabolism, such as chalcone isomerase (FtCHI), FtF3H, and FtF3. Other studies have shown that *Lepidium sativum* L. produces the highest total flavonoids under continuous 24 h light exposure (Ullah et al. 2019). In ginseng leaves, flavonoid content was found to be highest and of optimal quality under a 14-h/10-h (day/night) photocycle (Yan et al. 2020).

## Effect of light intensity on metabolism of pigment substances in food crops

Higher light intensity can stimulate the increase of carotenoids in food plants (Metallo et al. 2018), as plants accumulate more carotenoids in high light to reduce the damage caused by high light stress (Havaux 2014). At a photosynthetic photon flux density (PPFD) of 300 to 400 µmol m^−2^ s^−1^, the carotenoid content in cabbage increased with increasing light intensity (Brazaityte et al. 2016b, a). Supplementation with red or yellow light for 4 h per day significantly increased the carotenoid content in cucumbers (Cui et al. 2009).

However, high light intensity decreased the content of Violaxanthin in mustard microgreens (Kopsell et al. 2012). This is because under high light intensity, plants regulate photosynthesis through the xanthophyll cycle or violaxanthin cycle to reduce the damage caused by excess energy. Under strong light, violaxanthin is converted to zeaxanthin via antheraxanthin through a de-epoxidation reaction, transforming the absorbed high-energy light into heat energy and dissipating it. After the alleviation of photoinhibition, the cycle enzyme antheraxanthin epoxidase (ANT) re-epoxidizes ZEA back to violaxanthin (VIO), thereby enabling the adaptation to light energy. As comparison to 8%, 16% and 25% blue light, it was shown that 33% blue light was able to increase the content of carotenoids (lutein, neoxanthin, purpurin, as well as α-carotene and β-carotene) in mustard, sugar beet and parsley microgreens. Studies by Samuoliene et al. (2013) showed that the content of chlorophyll and carotenoids in romaine lettuce increased with increasing light intensity.

Excessive light intensity can lead to an increase in the content of compounds that are directly stimulated by light absorption (phenols, carotenoids) in green vegetables or indirectly affect compounds via metabolic pathways (ascorbic acid, tocopherols), thereby increasing their antioxidant capacity. In wheat, a blue:red ratio of 1:5 and LED treatment with high light intensity resulted in higher carotenoid content compared to low LED light intensity (Monostori et al. 2018). The authors suggest that high light intensity may lead to lower accumulation of chlorophyll, but at the same time to higher efficiency and thus greater biomass accumulation. Concomitant with changes in chlorophyll content, there are also corresponding changes in carotenoid content. This trend is related to a protective mechanism in plant leaves that protects them from photobleaching caused by intense light.

In summary, light intensity is a decisive factor influencing the synthesis of pigments in food plants. The pigment content of most food plants tends to increase with high light irradiation.

## Molecular Mechanisms of LED Light Quality Regulation on Plant Pigment Synthesis

**Fig. 2:**
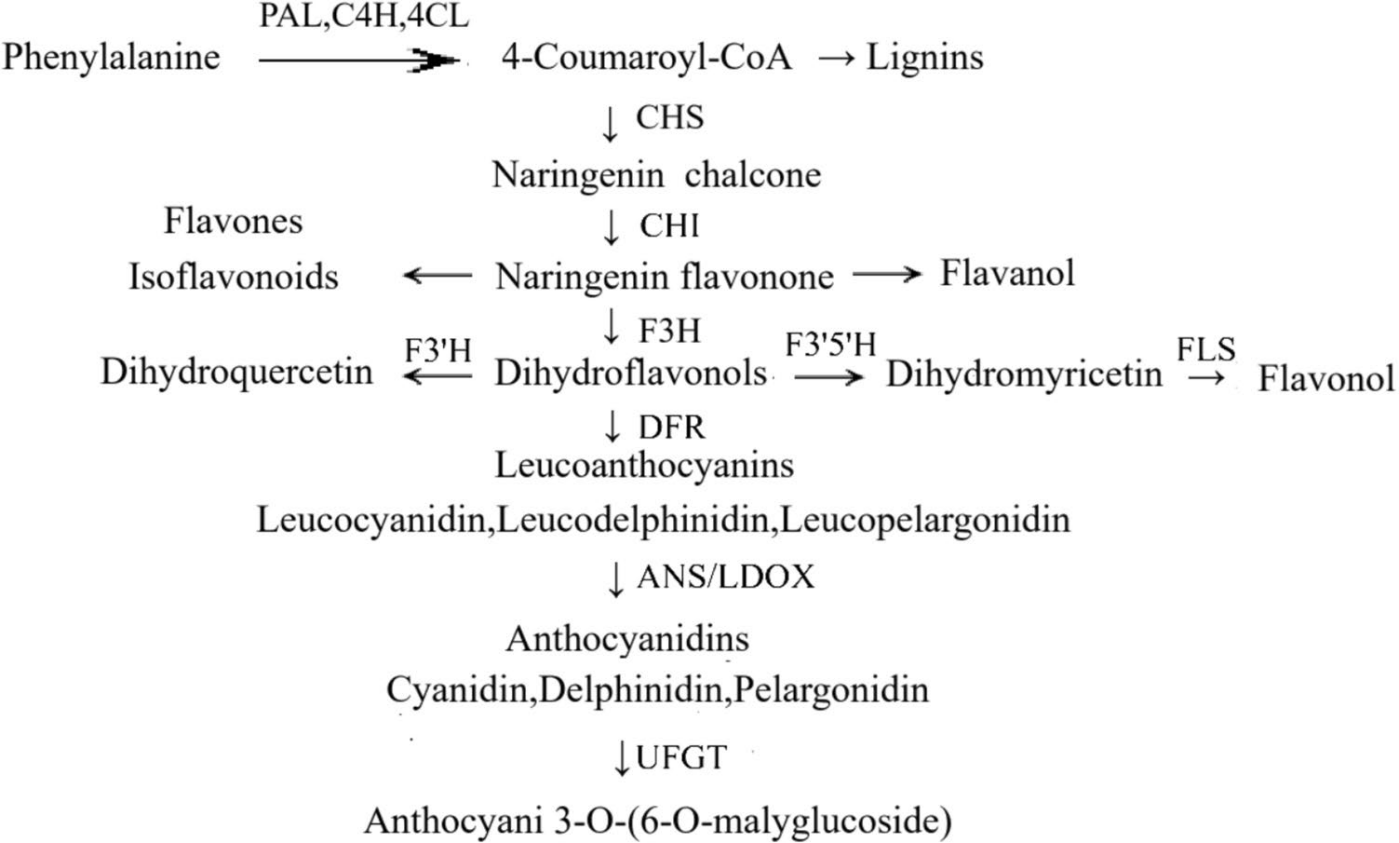
The synthetic pathway of anthocyanins

There are four types of photoreceptors in plants: phytochromes, which perceive red/far-red light (Li et al. 2011); cryptochromes and phototropins, which perceive blue/UV-A light (Kharshiing and Sinha 2015); and ultraviolet resistance locus 8 (UVR8), which perceives UV light (Heijde and Ulm 2012). Cryptochromes further influence the biosynthesis of carotenoids, anthocyanins, and chlorophyll (Lopez et al. 2012). The anthocyanin biosynthesis pathway in crops is illustrated in Fig. 2. Studies on grapes have shown that blue LED irradiation enhances the expression of myeloblastosis anthocyanin regulatory protein 1–2 (VlMYBA1-2), VlMYBA2, and UDP-glucose flavonoid 3-O-glucosyltransferase (VvUFGT), thereby promoting anthocyanin accumulation (Rodyoung et al. 2016). Red LED irradiation upregulated the expression of Md-MYB10 and UDP-glucose flavonoid 3-O-glucosyltransferase (MdUFGT) genes, which are related to anthocyanin biosynthesis in apples, leading to increased anthocyanin concentration (Lopez et al. 2012). Furthermore, irradiation of grapes with both blue and red LEDs upregulated the expression of genes related to anthocyanin biosynthesis, such as MYB transcription factor genes (Koes et al. 2005). Gene expression analysis revealed that shading significantly reduced anthocyanin accumulation in soybean sprouts, which was attributed to the downregulation of anthocyanin biosynthesis genes (GmDFR, GmANS, and GmUFGT) (Su et al. 2017).

**Fig. 3:**
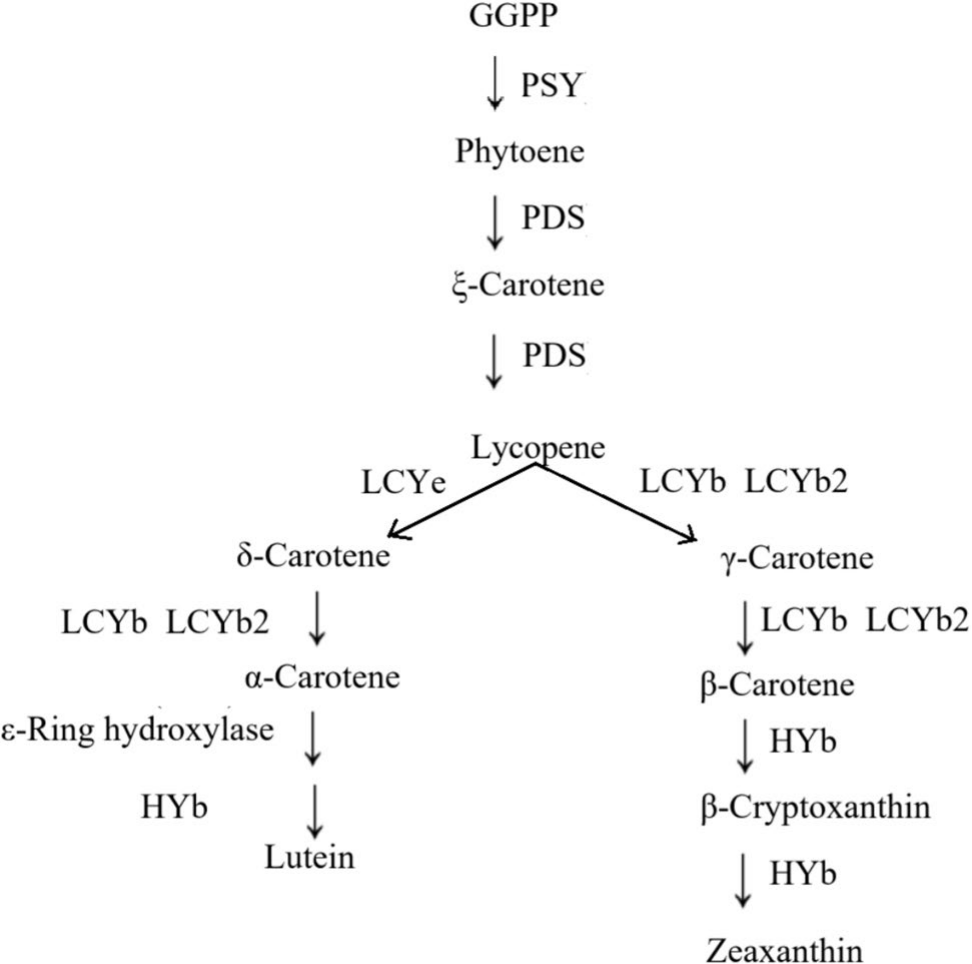
The synthetic pathway of carotenoids

The carotenoid biosynthesis pathway in food crops is illustrated in Fig. 3. White LED light promotes carotenoid biosynthesis by upregulating the expression of multiple carotenogenic genes, including FtPSY, FtLCYB, Lycopene ε-cyclase (FtLCYe), β-carotene hydroxylase (FtCHXB), ε-carotene hydroxylase (FtCHXE), and FtZEP (Tuan et al. 2013).Moreover, red light promoted the expression of carotenoid biosynthesis genes (CitPSY, CitPDS, CitZDS, CitLCYb1, CitLCYb2, and CitCHYb below 100B), leading to an increase in β-carotene content in Satsuma mandarin. Red LED irradiation could also reverse the negative effect of ethylene on lutein accumulation by upregulating the expression of citPSY, citPDS, citZDS, carotenoid isomerase (citCRTISO), citLCYb1, citLCYb2, citLCye, citCHYb, and citZEP, ultimately resulting in the accumulation of β-cryptoxanthin, all-trans violaxanthin, and lutein (Ma et al. 2015). Some studies have shown that blue light stimulates the synthesis of flavonoids by upregulating the expression of genes encoding PAL, CHS, and DFR. Furthermore, increasing blue light intensity from 50 μmol·m⁻^2^·s⁻^1^ to 100 μmol·m⁻^2^·s⁻^1^ shifts the synthesis of carotenoids from the β,β-carotene branch to the β,ε-carotene branch, thereby altering the ratio of β,ε-carotene to β-carotene in citrus fruits (Zhang et al. 2015).

**Fig. 4:**
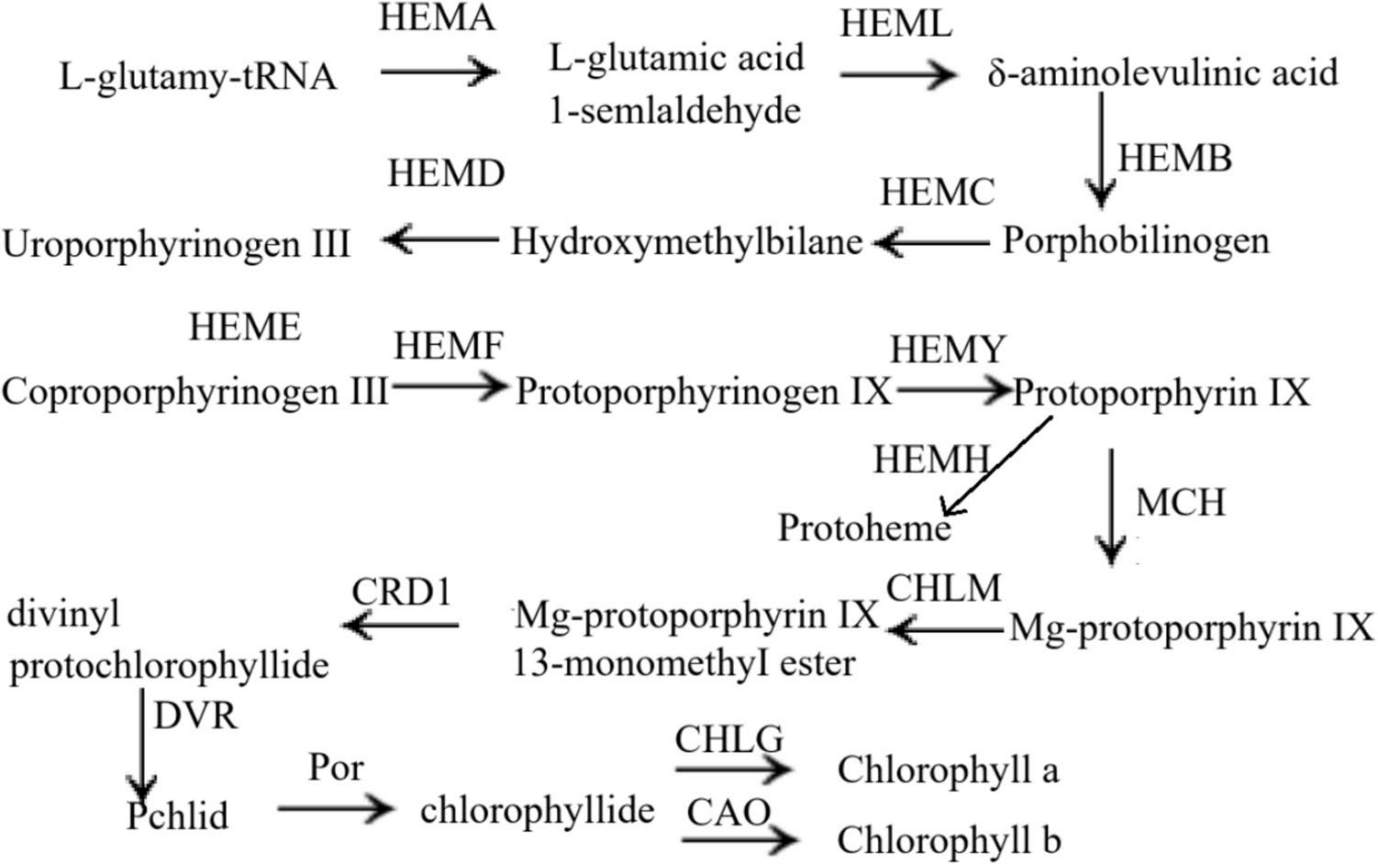
The synthetic pathway of chlorophyll

Blue light not only promotes chlorophyll synthesis by upregulating genes involved in chlorophyll biosynthesis, such as HemL/HemC/HemE/HemY/magnesium-protoporphyrin-O-methyltransferase, protochlorophyllide reductase, and chlorophyll(ide) b reductase (Li et al. 2017). However, red light is detrimental to chlorophyll formation, as it reduces the tetrapyrrole precursor δ-Aminolevulinic acid (ALA) in plants, thereby inhibiting chlorophyll production (Fan et al. 2013). The chlorophyll synthesis pathway in crops is illustrated in Fig. 4.

Under excessively high light intensity, plants produce large amounts of carotenoids to safeguard themselves from photodamage. On the one hand, carotenoids can act as a barrier for chlorophyll, allowing it to avoid excess light and prevent the formation of reactive oxygen species (ROS) (Loconsole et al. 2019). On the other hand, lutein provides photoprotection through non-photochemical quenching (NPQ). In this process, violaxanthin, antheraxanthin, and zeaxanthin epoxide are converted to zeaxanthin and lutein. These converted xanthophyll isomers effectively quench excess excitation energy in chlorophyll molecules, dissipating it as heat. These transformations of lutein alter the thylakoid membrane structure, interrupting the energy transfer from Photosystem II (PSII) to the reaction center, thus safeguarding the photosynthetic system (Loi et al. 2021).

## Mechanistic insights into the differential effects of LED light on pigment accumulation in various food crops

Different plant species contain different types of pigments, which can absorb light of different wavelengths. For example, chlorophyll mainly absorbs blue and red light, while carotenoids can absorb blue and green light. This diversity of pigments allows plants to utilize light energy in different ways when exposed to the same light source (Hatier et al. 2013).

Differences in photoreceptor sensitivity and signalling transduction pathways between different plant species can lead to different responses to LED light in terms of total pigment content (Massa et al. 2008). Different plant species show different responses of pigment biosynthesis genes to light induction, resulting in different pigment contents. In light-insensitive strawberries, for example, no significant differences in the expression of the genes for ANS, DFR and CHS were found between shaded and illuminated parts of the same fruit. However, in light-sensitive strawberries, the expression of CHS and ANS was significantly lower in the shaded parts than in the illuminated parts, resulting in lower anthocyanin accumulation (Zhou 2004).

The leaf structure and physiological characteristics of different plants can significantly influence the efficiency of light uptake and utilisation (Massa et al. 2008). For example, C4 plants with a higher chlorophyll content and better stomatal regulation can utilise LED light sources more effectively, thereby promoting pigment synthesis. Several metabolic pathways are involved in pigment biosynthesis, and the activities of key enzymes in these pathways can vary between different species (Zhu et al. 2008). These differences result in different pigment content under identical light conditions (Massa et al. 2008).

## LED light affects the expression of non-coding genes, and thus affects the metabolism of pigments

In *Brassica rapa*, irradiation with blue LED light decreased the transcript levels of miR156 and miR157, reducing the inhibition of their target genes squamosa promoter binding protein-like 9 (SPL9) and squamosa promoter binding protein-like 15 (SPL15), which ultimately promoted anthocyanin accumulation (Zhou et al. 2016). Blue light also indirectly affected the expression of phenolic compound-related genes in barley by regulating miRNA expression of the 156, 858 and 828 families, a regulatory mechanism that plays a role in controlling anthocyanin synthesis (Pech et al. 2022). Li et al. (2018) reported that blue light promotes the accumulation of flavonoids and epicatechin in longan by suppressing the expression of miR394 while inhibiting the synthesis of rutin. It was because blue light promoted the expression of its target genes transport inhibitor response 1–3 (DlTIR1-3), aluminum-activated malate transporter 12 (DlALMT12), and ADP-glucose pyrophosphorylase small subunit 1 (DlAPS1) by inhibiting the expression of miR393, miR394, and miR395.

On the one hand, these target genes positively regulated the expression of the most important flavonoid metabolism genes DlCHS, DlCHI, flavonoid 3'-hydroxylase (DlF3'H), DlDFR and leucoanthocyanidin reductase (DlLAR), which ultimately led to the accumulation of flavonoids. On the other hand, they negatively regulated the expression of the gene flavonol synthase (DIFLS) and thus inhibited the synthesis of rutin. Similarly, Dong et al. (2020) found that blue light treatment significantly downregulated the levels of microRNA 156e-3p (sly-miR156e-3p), microRNA 156e-5p (sly-miR156e-5p) and microRNA 394-3p (sly-miR394-3p) in tomato leaves.

Blue and UV-A light can downregulate the expression of miR156/157 in *Arabidopsis thaliana* and thereby increase the transcript abundance of its target genes SPL9 (Bra004674) and SPL15 (Bra003305). These SPL genes encode various regulatory molecules involved in regulatory processes, including the regulation of secondary metabolism in *Arabidopsis thaliana*, particularly the biosynthesis of anthocyanins (Wu et al. 2009). miR156 targeted SPL9 and competed with transparent testa 8 (TT8) for binding to production of anthocyanin pigment 1 (PAP1), disrupting the stability of the MYB-bHLH-WD40 complex. As a result, the expression of anthocyanin biosynthesis genes was suppressed, leading to negative regulation of anthocyanin accumulation (Cominelli et al. 2008). Zhou et al. (2016) showed that blue and UV-A light can induce the activation of genes involved in light signalling transduction, some of which promote anthocyanin biosynthesis. When the plants were exposed to UV or blue light, the transcription of miR156 and miR157 decreased, resulting in less inhibition of their target genes SPL9 and SPL15 and consequently promoting anthocyanin biosynthesis. This anthocyanin enrichment also triggered a feedback loop mediated by miR156/157 to maintain metabolic homeostasis.

In *Brassica rapa*, microRNA 828 (BrmiR828) negatively regulates the expression of the anthocyanin biosynthesis genes BrPAP1, BrMYB828 and trans-acting siRNA gene 4 (BrTAS4) by binding to mRNA and truncating some sequences. Under UV and blue LED irradiation, the expression of BrmiR828 was downregulated in *B. rapa*, leading to the accumulation of anthocyanins in young seedlings (Zhou et al. 2020). And Pashkovskiy et al. (2021) suggested that the increased accumulation of anthocyanins and total phenolics under red light induction may be due to the expression of ath-miR395a and the decrease in miR827 expression (Pashkovskiy et al. 2021). LED red light can promote the expression of miR156 gene and reduce the inhibitory effect of SPL protein on anthocyanins (Gulyás et al. 2023).

**Fig. 5:**
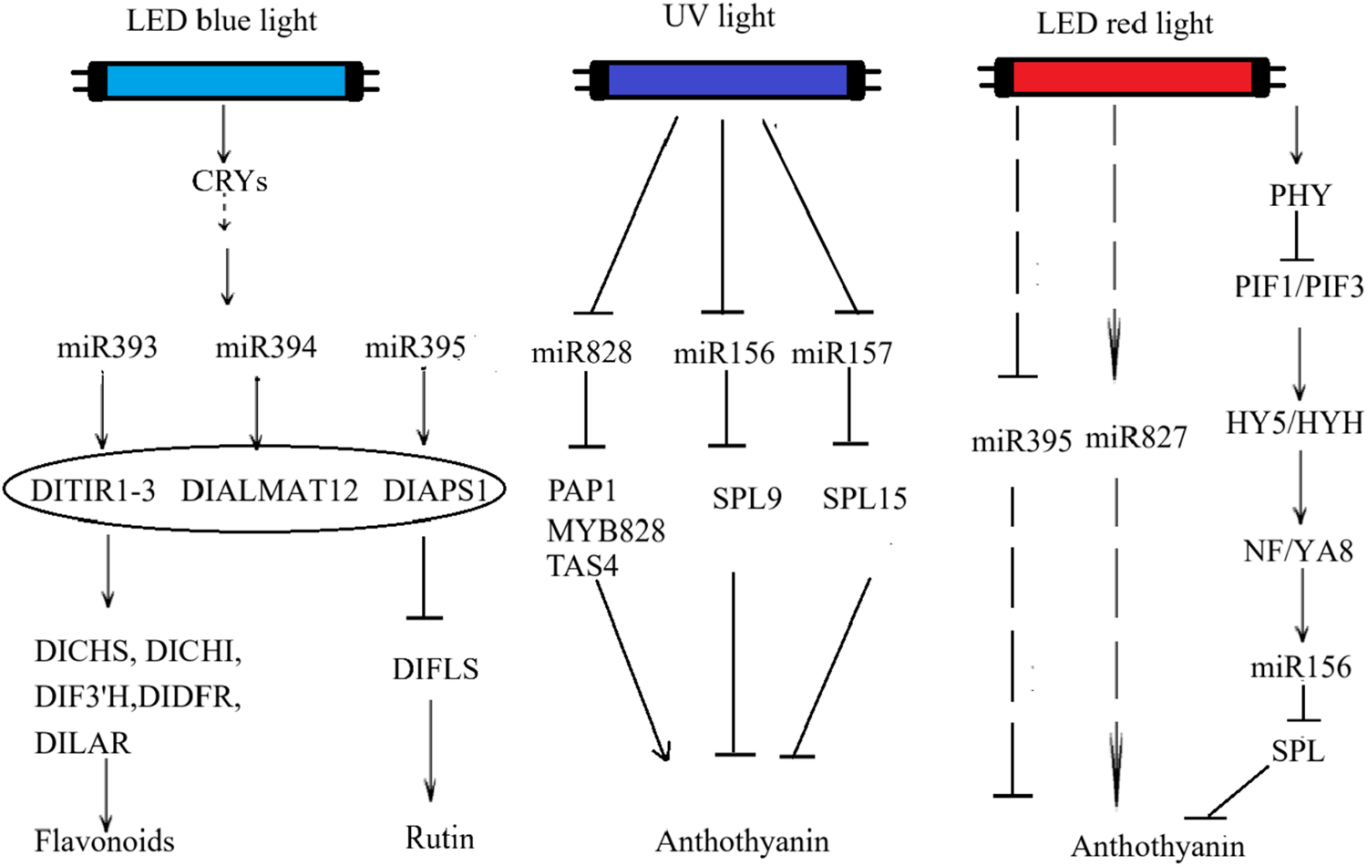
LED light regulates the pigment content in food crops by modulating non-coding RNA

Exposure to red LED increased the expression of genes of the chlorophyll synthesis pathway. Eight of these genes were found to be target genes of 118 long-chain non-coding RNAs (LncRNAs), and these LncRNAs could influence the expression of these genes through positive regulation (Yan et al. 2022). White light treatment promoted the yellowing process of *Arabidopsis thaliana* and induced the expression of miR157, miR163 and miR398, while it inhibited the expression of miR408, miR822 and miR834 (Choi et al. 2020). Figure 5 summarizes the regulatory effects of LED light on non-coding RNAs in food crops, thereby modulating pigment metabolism. The specific regulatory details of light on non-coding genes in several food crops are presented in Table 2.

**Table 2:** Effects of LED lights on the expression of non-coding genes related to pigment metabolism

No	Non-coding genes	LED light	Species	Modulation	References
1	miR156, miR157 ↓	Blue	*Brassica rapa*	Anthocyanins ↑	Zhou et al. (2016)
2	118 LncRNAs target and regulate 8 genes related to chlorophyll synthesis	Red	Broccoli	chlorophyll synthesis expression ↑	Yan et al. (2022)
3	miRNA 156 ↓	Blue	Barley	Anthocyanin ↑	Pech et al. (2022)
4	miR157, miR163, and miR398 ↑, miR408, miR822, and miR834 ↓	White	*Arabidopsis thaliana*	Yellowing process ↑	Choi et al. (2020)
5	miR156/157 ↓	Blue, UV-A	*Arabidopsis thaliana*	Anthocyanins ↑	Wu et al. (2009)
6	miR394	Blue	Longan	Flavonoids and epicatechin ↑ rutin ↓	Li et al. (2018)
7	sly-miR156e-3p, sly-miR156 e-5p, and sly-miR394-3p ↓	blue	Tomato		Dong et al. (2020)
8	BrmiR828 ↓	UV, blue	*Brassica rapa*	anthocyanins ↑	Zhou et al. (2020)

^↑^^: The increase in gene or metabolite content; ↓: The decrease in gene or metabolite content^

## Conclusions

Food crops are the foundation for human survival, and the pigments in food crops play a crucial role in maintaining human health, preventing chronic diseases, etc. Light is a key factor influencing the metabolism of pigments in crops. Blue light upregulates key enzyme genes, promoting flavonoid, anthocyanin, and carotenoid accumulation in most food crops, while red light enhances carotenoids by activating specific photoreceptors and regulating related gene expression. Specific photoperiods influence particular pigments, for instance, long photoperiods can promote the accumulation of specific flavonoids such as rutin. High light intensity generally enhances carotenoid accumulation, while potentially exerting a negative effect on chlorophyll content. Non-coding RNAs (ncRNAs) can influence pigment biosynthetic pathways by targeting and regulating the expression of crucial genes. Similarly, certain miRNAs can also directly participate in the regulation of pigment metabolism. Reasonable use of LED light sources can increase the pigment content in food crops, which plays an important role in improving the appearance of food crops and increasing the nutritional value of food. However, research on the effect of LED light on pigments in food crops is still at an early stage, and a systematic summary of the regulatory mechanism is lacking. This article provides an overview of the effect of LED light on the content of pigments in food plants and the underlying non-coding RNA regulatory mechanism, laying the foundation for application research of LED light in food production. Future research will integrate high-throughput omics and computational biology to dissect how different light sources or spectrums regulate plant hormone metabolites, related genes, and ncRNAs. This will clarify their roles in complex pigment metabolism and signaling, aiming for efficient pigment regulation and improved crop quality.
